# The Generic Conspiracist Beliefs Scale-5: further psychometric evaluation using a United Kingdom-based sample

**DOI:** 10.3389/fpsyg.2023.1303838

**Published:** 2023-11-29

**Authors:** Neil Dagnall, Andrew Denovan, Kenneth Graham Drinkwater, Alex Escolà-Gascón

**Affiliations:** ^1^Department of Psychology, Manchester Metropolitan University, Manchester, United Kingdom; ^2^Department of Quantitative Methods and Statistics, Comillas Pontifical University, Madrid, Spain

**Keywords:** Generic Conspiracist Beliefs Scale, conspiracy theories/ideation, brief measure, psychometric evaluation, scale evaluation

## Abstract

The 5-item Generic Conspiracist Beliefs Scale (GCB-5) is an abridged version of the 15-item GCBS. It was developed as a global measure of the tendency to engage in non-event-based, conspiracy-related ideation. The GCB-5 is appealing to researchers because of its brevity, which facilitates the measurement of belief in conspiracies alongside multiple constructs and/or in situations where resources are limited (time, etc.). Noting that several studies failed to find an adequate unidimensional fit in the parent GCBS measures across different contexts, the present study further assessed the psychometric properties of the GCB-5. This was necessary since the GCB-5 was validated using North American samples. Thus, to ensure that the GCB-5 was satisfactory for use with samples in the United Kingdom (UK), GCBS/GCB-5 items were administered to a large, representative UK-based sample (*N* = 1,331), alongside a range of validated conspiracy scales. Confirmatory factor analysis found that a one-factor GCB-5 model produced a good model fit. This specified that the GCB-5 was underpinned by a single dimension. Furthermore, the performance of the GCB-5 was equivalent to the longer GCBS. Both instruments produced similar mean item scores and standard deviations and were comparably positively correlated with concurrent measures. Although the GCB-5 internal reliability was lower than the GCBS, it was good. The GCB-5 also demonstrated configural, metric, and scalar invariance (among gender and age subgroups). This indicated that the GCB-5 was interpreted similarly by men and women and different age groups. Overall, results supported the assertion that the GCB-5 is a psychometrically satisfactory global measure of non-event-based, conspiratorial ideation.

## Introduction

The academic study of conspiracy is important because theories, despite being typically false, possess the potential to influence mainstream moods, opinions, and behaviors (Sunstein and Vermeule, 2009). This is especially true when conspiracies persist despite the existence of contradictory evidence and are endorsed by significant numbers of people (Irwin et al., 2015). In such circumstances, conspiracy theories can have harmful socio-political effects such as reducing faith in democratic processes, cultivating radical/extremist views, and undermining important official communications (i.e., guidance, campaigns, and initiatives) (Dagnall et al., 2020; Drinkwater et al., 2021, 2023). Although this negative conceptualization of conspiracies is overly simplistic since theories can perform beneficial functions (uncover official deception, reveal abuse of power, etc.), the majority of conspiracies nonetheless are false.

In this context, there is an important distinction between conspiracies originating from specious or erroneously interpreted information (e.g., COVID-19 was caused by 5G cellular networks) and theories based on truth (e.g., Watergate and Operation Northwoods) (Rastmanesh et al., 2023). Focusing on the fact that the majority of conspiracies are untruthful, the psychological investigation focuses predominantly on the detrimental effects of belief. Commensurate with this perspective, many studies focus on the notion that endorsement of conspiracies is associated with negative individual characteristics (flawed, biased thinking, skewed worldview, etc.; Drinkwater et al., 2012; Dagnall et al., 2015, 2017) and social factors (Douglas et al., 2019).

As scholarly interest in the domain evolved, investigators developed self-report instruments to assess belief in conspiracies. Prior to the emergence of scales assessing general ideation (see Brotherton and French, 2014), these were typically centered on real-life situations/events theories (Swami et al., 2017; Drinkwater et al., 2020; Kay and Slovic, 2023). This “theory-based” approach is derived from the supposition that belief in conspiracies is monological (Goertzel, 1994), whereby endorsement of one theory predicts advocacy of others (Sutton and Douglas, 2014). While there is evidence to support this view (Swami et al., 2011; Drinkwater et al., 2012), critics contend that it is oversimplified since endorsement is influenced by myriad variables. These include variations in belief as a function of topic (inter-category) and instance (intra-category). For example, though conspiracies about famous deaths are more strongly endorsed than alien cover-ups (inter-category), not all theories about famous deaths are validated equally (intra-category) (see Brotherton and French, 2014). Thus, although the theory-based approach possesses face validity, the extent to which responses to particular theories adequately sample construct domains and generalize across studies is debatable (see Hagen, 2018). Furthermore, theory validation is susceptible to cultural, historical, temporal, and economic influences. The presence of such contextual effects suggests that the theory-based approach provides only a limited, variable snapshot of belief.

Acknowledging these issues, Brotherton and French (2014) proposed that conspiracy advocacy was best assessed via general, abstract suppositions. These are non-event-based ideas, such as governments and scientists deceiving the general population. The advantage of this approach is its ability to measure beliefs about the typicality of real-world conspiratorial activity without contextual references. Moreover, by sampling a range of universal assumptions from which specific conspiracy theories arise, the “generic approach” possesses good content validity. This accords with Imhoff et al. (2022), who contend that there are important differences between worldview (conspiracy mentality) and specific beliefs (conspiracy theory). The former is more stable, less influenced by additional ideological content, and more normally distributed.

Recognizing the advantages of the generic (vs. theory) approach, Brotherton and French (2014) created the Generic Conspiracist Beliefs Scale (GCBS). Development and validation of the GCBS occurred through four studies. The initial study generated a pool of 75 items, which reflected broad (i.e., non-event-based) conspiracist claims. The content was derived from an examination of academic and popular literature. To ensure items were generic, the researchers used non-specific descriptors (“government,” “organizations,” etc.) in preference to definite entities/occurrences. These were administered to a sample of volunteers recruited via a blog post on Psychology Today and a public email list called Psychology of the Paranormal. Exploratory factor analysis reduced the item pool to 59 items, which loaded on five factors, namely, government malfeasance (GM; criminal conspiracy within government), extraterrestrial cover-up (ET; deceiving the public about alien existence), malevolent global conspiracies (MG; the notion that secret groups control world events), personal wellbeing (PW; concern about personal health and liberty), and control of information (CI; manipulation and suppression of information).

The second study created the 15-item GCBS by generating three items for each of the five factors. Subsequent assessments of the data using maximum likelihood confirmatory factor analysis confirmed that items best fit a five-factor correlated (vs. unidimensional) model. The emergent scale demonstrated reliability (i.e., internal and test-retest) and criterion-related validity (scores positively correlated with other measures of conspiratorial belief: Belief in Conspiracy Theories Inventory, BCTI, Swami et al., 2010; 9/11, Swami et al., 2010; 7/7 Swami et al., 2011; and Fictitious Red Bull, Swami et al., 2011).

Since the second study used a sample of university undergraduate students, the third and fourth studies assessed GCBS validity using a non-student sample of volunteers. The analysis found that GCBS scores correlated strongly with the BCTI (criterion-related validity) and belief in the paranormal and were moderately related to delusional ideation, higher anomie, and lower interpersonal trust (convergent validity). The pattern of correlations observed between the GCBS and study variables was similar to those produced by the BCTI. The final study established that the GCBS possessed discriminatory validity by demonstrating that scores were not related to extraversion, neuroticism, sensation seeking, or emotional intelligence. Despite reporting a superior model fit for the correlated five-factor model (vs. one-factor solution), Brotherton and French (2014) recommended using the total score because it captures a coherent set of allied beliefs that best reflect assumptions about the typicality of conspiratorial activity. While several studies have reproduced the correlated five-factor model (i.e., Siwiak et al., 2020; Fasce et al., 2022), others have reported alternative solutions and found a poor fit for a single-factor solution (e.g., Swami et al., 2017; Atari et al., 2019; Majima and Nakamura, 2020).

Noting failures to replicate the correlated five-factor solution, Drinkwater et al. (2020) further examined the psychometric properties of the GCBS. They compared a university-based sample with data collected by a market research company. The correlated five-factor solution (vs. one-, two-, and three-factor models) produced superior fit, and outcomes were invariant across groups. Factor correlations specified a strong degree of relationship, which was representative of generalized conspiracist suppositions. Moreover, summative and subscale scores demonstrated good internal reliability and convergent validity (correlations with proneness to reality testing deficits and cognitive insight, signifying decreased critical and higher levels of subjective-intuitive thinking) (Drinkwater et al., 2020).

Overall, results across studies suggest that whilst the GCBS is generally robust, its factorial structure may be prone to contextual variation. Additionally, the poor fit of the unidimensional solution indicates that, though strongly related, GCBS factors vary in level of endorsement. For instance, in study 2 of the Brotherton and French (2014) article, GM shared greater variance with MG (66%), PW (75%), and CI (55%) than ET (31%). This variability illustrates that not all factors are readily endorsed. The poor fit of the one-factor solution is problematic because studies typically use the GCBS as a global measure (e.g., Marchlewska et al., 2022; Cosgrove and Murphy, 2023; Harmon-Jones and Szymaniak, 2023). Moreover, Kay and Slovic (2023) have proposed a concise (i.e., 5-item) unidimensional version of the GCBS, the GCB-5.

Due to the fact that belief in conspiracies is often assessed alongside myriad other constructs within lengthy test batteries, the existence of a brief, equivalent measure has significant practical and logistical advantages. These include reduced cost, time, and cognitive load placed on respondents. Survey length is a crucial consideration since recruitment costs increase as a function of item number and complexity. In terms of time, longer testing sessions normally result in higher levels of drop-out (withdrawal) and non-completions (e.g., increased potential for interruptions). Furthermore, with university participation pools, students completing longer surveys receive more credits, meaning that they are less inclined to participate in subsequent research. Regarding cognitive load, longer testing sessions are more likely to produce inattention and/or careless responses, reducing validity and reliability.

The GCB-5 was developed and psychometrically evaluated through five studies (Kay and Slovic, 2023). In study 1, participants completed the GCBS, and the highest loading items from each of the factors were identified; these corresponded with Brotherton and French (2014). This procedure ensured that the GCB-5 sampled the same domains as the GCBS. Within study 1, GCB-5 reliability was evaluated by examining factor structure, internal consistency, and criterion validity (i.e., the tendency to concurrently endorse other measures of conspiracist ideation). Study 2 further evaluated the construct validity of the GCB-5 via consideration of relationships with convergent (e.g., delusional ideation) and divergent (e.g., trustworthiness) measures. Studies 3 and 4 extended validation by introducing further variables (e.g., uniqueness) and informant reports (i.e., ratings of respondents by well-acquainted others) (Vazire, 2006).

Outcomes indicated that the GCB-5 (vs. GCBS) had comparable levels of criterion validity and greater criterion validity (as assessed by higher correlations with the BCTI) than the Conspiracy Mentality Questionnaire (Bruder et al., 2013). Finally, study 5 extended the evaluation of GCB-5 via analysis of its relationships with theoretically relevant social and political issues (e.g., support for stricter voting laws). This established that high scores on the GCB-5 were associated with greater acceptance of virtuous violence (i.e., hostile actions perceived as morally right). A limitation of Kay and Slovic (2023) was that studies 1 to 4 used the same undergraduate participant pool. Study 5 recruited participants from Prolific, a commercial provider of samples.

Noting that several studies have failed to find a good fit for a unidimensional GCBS solution, there has been some evidence of contextual variations, and that the GCB-5 was validated using only North American samples, the present study appraised the measure's psychometric properties using a large, representative United Kingdom-based sample. That is a sample with an equal gender balance, a wide age range, and a variety of occupations. This was important because psychological studies often employ university-based samples. These, as a consequence of the sample, characteristics are restricted in terms of age, vocation, education, etc. Hence, the use of a broad, general sample enhanced finding extrapolation, increasing scale applicability.

In addition to model fit, internal reliability, convergent validity (via comparison of the GCB-5 and GCBS with concurrent indexes of belief in conspiracies), and invariance testing (age and gender) were undertaken. The purpose of these analyses was to establish whether researchers could effectively use the GCB-5 (vs. GCBS) as a brief measure of generic conspiracy beliefs in UK general samples.

## Methods

### Participants

The sample comprised 1,331 participants [mean age (*M*_*age*_) of 44.97, *SD* = 12.64, and a range of 18–70]. Regarding gender, there were 668 (50.19%) men (*M*_*age*_ = 47.02, *SD* = 11.88, and range of 18–69), 658 (49.44%) women (*M*_*age*_ = 42.82, *SD* = 13.01, and range of 18–70), and 5 (0.38%) individuals who preferred not to say (*M*_*age*_ = 54.60, *SD* = 12.42, range 34–66). Participants were UK-based, with a minimum age of 18 years, recruited by Bilendi, an acknowledged provider of good-quality, representative online samples (Salak et al., 2021) that are equivalent to those collected by traditional methods (Kees et al., 2017).

### Measures

#### Generic Conspiracist Beliefs Scale: GCB-5

Generic Conspiracist Beliefs Scale (Brotherton and French, 2014) assesses general conspiratorial ideation. It is composed of 15 items, which are presented as statements (e.g., “A small, secret group of people is responsible for making all major world decisions, such as going to war”). Participants respond by completing a 5-point Likert-type scale (1 = definitely not true to 5 = definitely true). The scale comprises five subscales (see section Introduction). Item summation produces an overall total, with higher scores being indicative of greater levels of generic conspiratorial ideation. The GCB-5 5 (Kay and Slovic, 2023) comprises the highest loading item from each factor, these are totalled to produce a global score (see Kay and Slovic, 2023).

### Concurrent measures

#### Conspiracy Mentality Scale

The Conspiracy Mentality Scale (CMS) (Imhoff and Bruder, 2014) contains 12 items that conceptualize conspiracy as a generalized political attitude distinct from established views about government (e.g., social dominance orientation). Within the CMS, items appear as statements (i.e., “There are many very important things happening in the world about which the public is not informed”). Respondents record their answers on a 10-point Likert-type scale ranging from 1 (extremely unlikely) to 10 (extremely likely). The summation of items produces an overall score. Higher scores denote a greater conspiracy mentality.

#### Beliefs in Conspiracy Theories

The Beliefs in Conspiracy Theories (BCT) (Leman and Cinnirella, 2013), via eight items, assesses belief in specific conspiracy theories based on real-world events/organizations. Items are displayed as declarations (e.g., “The American moon landings were faked”), and participants respond using a 5-point Likert-type scale, ranging from 1= strongly disagree to 5 = strongly agree. Totalling items produces an overall score, and higher scores indicate greater advocacy of real-world conspiracy theories.

#### Single-Item Conspiracy Belief Scale

Single-Item Conspiracy Belief Scale (SCBS) (Lantian et al., 2016) measures the general tendency to believe in conspiracy theories. The instrument asks participants, using a 9-point Likert-type scale (where 1 = completely false and 9 = completely true), to respond to the statement, “I think that the official version of events given by the authorities very often hides the truth.” Higher scores reflect a greater belief in conspiracies.

The CMS, BCT, and SCBS are established scales that conceptualize and assess belief in conspiracy theories in differing ways. They were selected for the present study because, collectively, they reflect the range of major measurement perspectives (i.e., CMS, generalized political attitude; BCT, specific theories; and SCBS, general tendency). Additionally, these instruments have been featured in peer-reviewed, published research and have attested to psychometric properties (see Leman and Cinnirella, 2013; Imhoff and Bruder, 2014; and Lantian et al., 2016, respectively).

### Procedure

Individuals who replied to the respondent call used a web link to access the Participant Information Sheet, which outlined the aims, objectives, and ethics of the study. Those wishing to participate clicked consent and advanced to the survey. This comprised a demographic section (i.e., preferred gender and age) and the measurement instruments. The scale presentation was randomized across participants to counter potential order effects. Once participants completed the survey, they were debriefed. Since the study used a cross-sectional design, procedural remedies to reduce common method variance, evaluation apprehension, and social desirability were employed (see Dagnall et al., 2022a,b). Specifically, both general and scale instructions emphasized the uniqueness of subsections. This created a psychological distance between scales and encouraged participants to reflect on their responses (Krishnaveni and Deepa, 2013). Additionally, participants were directed to carefully read statements, work at their own speed, respond to all items, and be aware that there were no incorrect answers.

### Ethics statement

The Manchester Metropolitan University Faculty of Health, Psychology, and Social Care Ethics Committee granted ethical approval (Project ID, 11440).

#### Analytical strategy

Following data screening, the GCB-5 structure was assessed using confirmatory factor analysis (CFA). A unidimensional solution was tested, followed by invariance comparing subgroups (gender and age quartile). Invariance analysis examined a series of progressively restrictive models and a test of the form (configural) prior to examining the equivalence of factor loadings (metric) and intercepts (scalar). Comparison of latent means (i.e., GCB-5 with GCBS; Conspiracy Mentality Questionnaire, CMS; Beliefs in Conspiracy Theories, BCT; and a single-item measure, SCBS) occurred prior to convergent validity testing.

CFA included the comparative fit index (CFI), Tucker-Lewis index (TLI), standardized root-mean-square residual (SRMR), and root-mean-square error of approximation (RMSEA). Good fit is indicated by CFI ≥ 0.95, TLI ≥ 0.95, SRMR ≤ 0.08, and RMSEA ≤ 0.06 (Hu and Bentler, 1999). Loadings ≥0.40 are adequate and representative of the factor(s) (Gliner et al., 2011). For invariance testing, alongside model fit, CFI and RMSEA changes verified the degree of equivalence between models. Respective CFI and RMSEA changes of ≤0.01 and ≤0.015 are satisfactory (Chen, 2007). In addition, a Satorra-Bentler chi-square difference test (S–B χ^2^) determined if model changes were significant.

## Results

### Data screening

Scrutiny of multivariate normality indicated non-normal data. Specifically, Mardia's kurtosis (*b2p*) = 38.96, *p* < 0.001, and Srivastava's skewness (*b1p*) = 3.70, *p* < 0.001. Accordingly, maximum likelihood with robust standard error (MLR) estimation was utilized.

### Confirmatory factor analysis

The one-factor model of the GCB-5 displayed good model fit: χ^2^ (5) = 17.87, *p* = 0.003, CFI = 0.99, TLI = 0.98, RMSEA = 0.04 (90% CI 0.02 to 0.06), and SRMR = 0.01. Assessment of factor loadings revealed an average loading of 0.71 (range of 0.61 to 0.77). These findings indicated that a single dimension underpinned the GCB-5. A test of gender invariance (i.e., men and women) revealed a good fit for the configural model (Table 1). Variations in CFI and RMSEA did not exceed 0.01 and 0.015 at the metric or scalar level and were supported by non-significant changes at each stage: configural vs. metric S–B χ^2^ (4) = 7.18, *p* = 0.126; metric vs. scalar S–B χ^2^ (4) = 6.07, *p* = 0.193. Invariance tests for age quartiles (i.e., 18–34, 35–46, 47–55, 56+) also demonstrated a good fit for the configural model and revealed no meaningful variation in CFI and RMSEA at the metric and scalar level. Non-significant changes existed, configural vs. metric S–B χ^2^ (12) = 11.37, *p* = 0.497; and metric vs. scalar S–B χ^2^ (12) = 18.14, *p* = 0.111. These results demonstrated invariance across gender and age quartiles.

**Table 1:** Fit indices for five-item GCBS invariance models.

**Model**	**χ^2^**	**S–B χ^2^**	***df***	**CFI**	**CFI difference**	**TLI**	**SRMR**	**RMSEA (90% CI)**
**Gender**								
Configural	20.82^*^		10	0.99		0.99	0.01	0.04 (0.02–0.06)
Metric	28.25^*^	7.18	14	0.99	0.002	0.99	0.02	0.04 (0.02–0.06)
Scalar	34.63^*^	6.07	18	0.99	0.001	0.99	0.03	0.04 (0.02–0.06)
**Age quartile**								
Configural	33.61^*^		20	0.99		0.98	0.01	0.04 (0.02–0.07)
Metric	46.37^*^	11.37	32	0.99	0.001	0.99	0.03	0.03 (0.01–0.05)
Scalar	64.43^*^	18.14	44	0.99	0.004	0.99	0.04	0.03 (0.01–0.05)

χ^2^, chi-square goodness-of-fit statistic; S–B χ^2^, Satorra-Bentler chi-square difference; df, degrees of freedom; CFI, comparative fit index; TLI, Tucker-Lewis Index; SRMR, standardized root-mean-square residual; RMSEA, root-mean-square error of approximation; ^*^χ^2^significant at p < 0.05.

Comparison of latent means (reference group men) revealed no significant gender difference (men vs. women) in GCB-5 scores, *M* = 0.06, *p* = 0.153, *d* = 0.07. For age, with the 18–34 quartile as the reference group, no significant difference existed in the GCB-5 scores for the 35–46 quartile, *M* = 0.10, *p* = 0.107, and *d* = 0.12. However, the 47–55 quartile demonstrated significantly lower GCBS scores than the 18–34 quartile, *M* = 0.31, *p* < 0.001, and *d* = 0.39 (large effect). The 56+ quartile also reported significantly lower GCBS scores than the 18–34 quartile, *M* = 0.33, *p* < 0.001, and *d* = 0.42 (large effect). The mean difference for this quartile was greater than the difference for the 47–55 group.

#### Convergent validity

Correlations of the GCB-5 with the GCBS, its subscales, and additional conspiracy scales (CMS, BCT, and SCBS) are displayed in Table 2. The results specified that the GCB-5 correlated strongly and significantly with the GCBS and its subscales (*r*s of 0.78 to 0.96). Strong and significant associations also existed between the GCB-5 and CMS, BCT, and the SCBS. Similar results existed for the GCBS. Moreover, correlation strength did not differ significantly. Explicitly, non-significant differences existed between the GCB-5 and GCBS with CMS (*z* = 1.58, *p* = 0.113), BCT (*z* = 1.94, *p* = 0.051), or the SCBS (*z* = 0.84, *p* = 0.399). Satisfactory omega reliability existed for all scales (see Table 2).

**Table 2:** Convergent validity of the five-item GCBS (GCB-5).

**Variable**	***M***	***SD***	**ω**	**1**	**2**	**3**	**4**	**5**	**6**	**7**	**8**	**9**	**10**
1. GCB-5	2.89	0.92	0.83		0.96^**^	0.84^**^	0.84^**^	0.78^**^	0.86^**^	0.78^**^	0.64^**^	0.72^**^	0.59^**^
2. GCBS	2.89	0.87	0.94			0.89^**^	0.87^**^	0.82^**^	0.89^**^	0.80^**^	0.67^**^	0.76^**^	0.61^**^
3. GM	2.89	1.04	0.84				0.73^**^	0.63^**^	0.76^**^	0.66^**^	0.60^**^	0.68^**^	0.58^**^
4. MG	2.94	1.05	0.87					0.60^**^	0.73^**^	0.67^**^	0.67^**^	0.63^**^	0.53^**^
5. ET	2.54	1.12	0.87						0.69^**^	0.51^**^	0.44^**^	0.70^**^	45^**^
6. PW	2.69	1.02	0.80							0.64^**^	0.56^**^	0.69^**^	0.51^**^
7. CI	3.37	0.88	0.73								0.63^**^	0.52^**^	0.54^**^
8. CMS	6.42	1.58	0.90									0.55^**^	0.64^**^
9. BCT	2.67	0.78	0.77										0.58^**^
10. SCBS	5.83	2.15	-										

GCB-5, 5-item Generic Conspiracist Beliefs Scale; GCBS, Generic Conspiracist Beliefs Scale; GM, government malfeasance; MG, malevolent global conspiracies; ET, extraterrestrial cover-up; PW, personal wellbeing; CI, control of information, CMS, Conspiracy Mentality Scale; BCT, Belief in Conspiracy Theories Inventory; SCBS, Single-Item Conspiracy Belief Scale.

** p < 0.001.

## Discussion

The present study, using a representative UK sample, found that the GCB-5 (Kay and Slovic, 2023) was a satisfactory measure of non-event-based, generic conspiracy-related ideation. Consistent with Kay and Slovic's (2023) analysis of North American samples, the GCB-5 fitted well to a one-factor model. This outcome concurred with the notion that the GCB-5 is a brief, unidimensional measure equivalent to the GCBS. Commensurate with this supposition, both instruments produced similar mean item scores and standard deviations. Furthermore, though GCB-5 (vs. GCBS) internal reliability was lower (ω = 0.83 vs. ω = 0.94), it was good. Reduced internal consistency frequently occurs when the length of a validated instrument is decreased because test developers typically refine scales to maximize internal reliability (Kemper et al., 2019). Hence, item removal increases sensitivity to random error (e.g., misinterpretation) (McCrae et al., 2011). The GCB-5 also demonstrated configural, metric, and scalar invariance (among gender and age subgroups). This indicated that the GCB-5 was invariant (i.e., interpreted similarly by men and women and different age groups).

The equivalent performance of the GCB-5 (vs. GCBS) showed that item selection was sound. Kay and Slovic (2023) constructed the GCB-5 by taking the highest loading items from each of the five GCBS factors. Analysis in the current article confirmed that these items were representative of domain content. This was further evidenced by similarly sized, positive relationships between the GCB-5, GCBS, and concurrent measures (CMS, BCT, and SCBS). Indeed, differences were minor, indicating that the GCB-5 produced comparable outcomes to the GCBS. Although the GCB-5 and GCBS were strongly correlated with concurrent measures, there remained a significant proportion of unexplained variance. In terms of commonality, the GCB-5/GCBS shared 41–45%, CMS; 52–58%, BCT; and 35–37% SCBS.

Collectively, these findings demonstrated that, although related, measures assessed different elements of conspiratorial belief/ideation. Accordingly, scores on the GCB-5/GCBS were below the mean, whereas scores on the CMS, which conceptualizes conspiracy as a generalized political attitude, were above the midpoint. Nuanced differences between scales imply that endorsement of conspiracies is best conceptualized as dialogical rather than monological. Therefore, subsequent investigations should carefully consider which operationalisation of conspiracism best suits their objectives. Particularly, one must be cognizant of distinctions between important terms such as belief, ideation, and mentality. These are often used interchangeably or without precise definitions, resulting in conceptual imprecision/obfuscation.

Though the GCB-5 has demonstrated good psychometric properties in both UK and North American samples, it is important to remember that the scale was designed as a brief general measure for inclusion in large test batteries. The GCB-5 is necessary because it provides investigators with a psychometrically validated, expedient index of generic conspiracy-related ideation. This is especially advantageous when researchers have limited testing time or resources and conspiratorial thinking is being assessed alongside multiple constructs. Explicitly, being a short scale, the GCB-5 places fewer demands on respondents. This enables researchers to assess conspiracy-related ideation in time-pressured real-life settings such as schools and with groups who possess lower or reduced cognitive abilities (e.g., adults with cognitive impairment). Furthermore, since the GCB-5 derives from the GCBS and produces equivalent general scores, its outcomes are directly comparable with pertinent previous work (i.e., research using similar samples) that has used the parent measure. This is highly beneficial because the GCBS is the most widely used measure of belief in conspiracies.

Despite these benefits, it is important to acknowledge that the GCB-5 provides only a global snapshot of conspiracy-related ideation. Due to its brevity, the GCB-5 is not sufficiently nuanced to detect differences between belief types. To examine differences in the composition of beliefs, multidimensional measures are required. This type of evaluation is necessary because, though participants may have similar levels of belief, the constituents of their ideation can vary significantly. This is a common problem with the variable-centered approach, where researchers implicitly assume that believers are a homogeneous population, as designated by scale scores (Drinkwater et al., 2022).

Noting this, subsequent research should employ a person-centered approach (e.g., latent profile analysis) that captures within-participant group variations (Bouckenooghe et al., 2019). Investigators can achieve this by combining GCB-5 scores with allied factors such as paranoia, schizotypy, and mistrust. Differences between emergent groups/classes are important as they will provide nuanced insights into deviations among conspiracy believers (Denovan et al., 2018). The brief nature of the GCB-5 will greatly facilitate this process by allowing investigators to include belief in conspiracies alongside multiple constructs.

Moreover, since ideas vary in terms of typicality and plausibility, it may be that only some cognitions are non-adaptive. With reference to the GCBS, previous research reports lower scores on the extraterrestrial cover-up. This suggests that these ideations are normatively less common and therefore potentially related to different psychological states than more commonly endorsed concerns. The usefulness of examining facets of conspiracy-related ideation is highlighted by cross-cultural comparisons, where beliefs are likely to vary as a function of societal, political, and historical factors. Indeed, previous research reports that belief in conspiracies is higher in historically traumatized societies (Bilewicz, 2022), particularly those with greater levels of collectivism, corruption, and lower gross domestic product (Hornsey and Pearson, 2022). This likely affects scores related to some aspects of ideation (e.g., political intrigue) more than others. Acknowledging this, further validation of the GCB-5 is necessary to ensure that it is invariant across national groups. Once invariance is established, it will be possible for researchers to draw meaningful cross-country comparisons (Plouffe et al., 2023) and identify particular cultural and social factors that influence belief in conspiracies.

A further limitation of the present study was that it was cross-sectional (i.e., data were collected at only one point in time). In this context, it is important that subsequent research establishes measurement stability over time (i.e., test-retest reliability) and concomitantly investigates longitudinal changes in belief as a function of political-social influences (i.e., government ratings and economic stability). Despite these concerns, the present study established that the GCB-5 was a psychometrically satisfactory global measure of non-event-based, conspiratorial ideation. A particular advantage of the GCB-5 is that the scale's brevity allows researchers to include the instrument within large test batteries.

## Data availability statement

The raw data supporting the conclusions of this article will be made available by the authors, without undue reservation.

## Ethics statement

The studies involving humans were approved by the Manchester Metropolitan University Faculty of Health, Psychology and Social Care Ethics Committee granted ethical approval (Project ID, 11440). The studies were conducted in accordance with the local legislation and institutional requirements. The participants provided their written informed consent to participate in this study.

## Author contributions

ND: Conceptualization, Methodology, Validation, Writing—original draft. AD: Conceptualization, Data curation, Formal analysis, Methodology, Writing—review & editing. KD: Data curation, Resources, Writing—review & editing. AE-G: Writing—review & editing.
